# Research on team efficacy in CBA games based on complex network theory

**DOI:** 10.1371/journal.pone.0344684

**Published:** 2026-03-31

**Authors:** Weishuai Guo, Rui Dong, Tengfei Dong, Ling Chen

**Affiliations:** 1 College of Physical Education, Pingdingshan University, Pingdingshan, Henan Province, China; 2 School of Physical Education and Sports Science, South China Normal University, Guangzhou, China; 3 Department of Physical Education, Jeonbuk National University, Jeonju, Republic of Korea; 4 Department of Leisure Sports, Hunan University of Medicine, Huaihua, Hunan Province, China; Universidade Estadual de Maringa, BRAZIL

## Abstract

Using complex network theory, we quantify the topological characteristics and anti-destruction resilience of the 12 playoff teams that contested the 2023 Chinese Basketball Association (CBA) season. By constructing similarity networks based on players’ performance indicators, this study examines various topological features, including node degree, betweenness centrality, clustering coefficient, and average path length, and assesses their robustness under random and deliberate attacks.The results indicate that the structural balance of the network exerts a significant influence on a team’s resilience. For example, the Liaoning Bensteel team exhibited high network connectivity and resilience, with an average node degree of 11, a clustering coefficient of 0.83, and an average path length of 0.72. In contrast, the Jiangsu Kendia demonstrated greater structural vulnerability when key nodes were compromised, possessing a maximum node degree of 10, but a clustering coefficient of only 0.74, leading to an increased average path length of 1.17. Furthermore, teams with high clustering coefficients, such as Shandong High-Speed, which achieved a coefficient of 0.82, exhibit close internal cooperation and efficient ball movement.Consequently, it is recommended that CBA teams enhance their resilience by improving the balance and redundancy of key nodes within their network structures, thereby providing a scientific basis for basketball tactical planning and team management. These findings offer a novel perspective on understanding and enhancing the organizational structure and competitive performance of basketball teams.

## Introduction

Basketball, a sport of global popularity, relies heavily on team collaboration [1]. Each player is required not only to exhibit individual skills but also to cooperate cohesively within the team [2]. Consequently, a systematic study of basketball can significantly enhance a team’s long-term performance and adaptability. Team efficacy reflects not only the composite performance of team members but also their capacity to adapt, maintain, and evolve—key factors in assessing the success of any sports team [3].

In recent years, complex network theory has emerged as a powerful tool for analyzing team structures and dynamics, particularly in the field of sports science [4]. By mapping player interactions and positional changes onto a network of nodes and edges, researchers can thoroughly investigate the structural characteristics and dynamic changes occurring within teams. For instance, studies conducted by researchers such as Manuel have employed social network analysis to examine the cooperative relationships and core roles of players across different age groups in basketball teams [5]. Their findings illuminate the intrinsic social structure of basketball and the complexities involved in tactical execution. Furthermore, research by scholars like Kempe [6], who have utilized Self-Organizing Maps (SOM) and Dynamic Controlled Neural Networks (DCNN) [7], has demonstrated how these technologies can effectively identify and respond to dynamic tactical changes, thereby providing valuable insights for coaches.

Despite these advancements, a significant gap remains in research regarding how team network structures in basketball respond to tactical changes and unexpected events. Existing studies often ignore the real-time changes in network topology during games and their direct effects on team performance. As basketball enters the so-called ‘small ball era,’ characterized by substantial shifts in technical and tactical styles and an accelerated pace of play [8], there is an increasing demand for the application of complex network approaches to thoroughly investigate each team’s offensive efficiency and network connectivity [9].

Although the NBA is more globally visible, the CBA offers three scientific advantages for a first-of-its-kind study: stylistic homogeneity—fewer mid-season roster changes and less variation in coaching philosophies reduce confounding factors; open data—league-wide box scores and play-by-play logs are available for every playoff game; and strategic relevance—China’s national sports program explicitly requests evidence-based roster construction.

Therefore, this study employs complex network theory to conduct a comprehensive analysis of the network structures of 12 teams in the 2023 CBA playoffs, with a specific emphasis on topological features such as node degree, betweenness centrality, and clustering coefficient. By constructing networks based on player performance similarities, we evaluate how these features influence the teams’ network robustness in the face of both random and deliberate attacks. This methodology not only enriches the tactical analysis of the CBA but also lays a scientific foundation for basketball training and match decision-making, thereby facilitating the integration of theory and practice in basketball and other sports. The findings of this research contribute to the optimization of tactical decisions and the enhancement of team collaboration efficiency, thereby paving new pathways for future research in sports science.

## Methods

### Complex network research methods

The study of complex networks originated from Erdős and Rényi’s research on random graphs in 1960 [10]. Since the discovery of small-world and scale-free properties, research in this field has accelerated significantly [11,12]. Today, complex networks are widely applied across various domains, including structural and dynamic systems, power networks, time series, and even clinical medicine, attracting considerable attention [13–17]. Through in-depth research of network structure, function, and dynamics, researchers aim to quantify network characteristics and simulate optimal network configurations, establishing this as a rapidly growing interdisciplinary field.

In the study of topology and resilience within CBA basketball teams, metrics such as node degree and node strength are extensively used to reflect the cooperative relationships and significance among team members [18,19]. These metrics facilitate the analysis of the internal topological structure of teams, thereby revealing their resilience against various challenges and changes. This study integrates complex network research methodologies tailored to the specific conditions of CBA basketball teams. By assigning appropriate weights, it quantitatively analyzes the teams’ topological features and resilience, providing a scientific basis for team management and decision-making, and promoting overall improvements in team performance.

### Construction of network models

This study constructs 12 similarity networks for the teams participating in the 2023 CBA Playoffs, to analyze topology and resilience, and perform a cross-sectional comparative study. To thoroughly evaluate each player, we selected the most crucial data metrics defined in Oliver’s research for basketball studies, known as “the Four Factors”: effective field goal percentage (eFG%), free throw attempt rate (FTA%), turnover ratio (TO%), and offensive rebounding percentage (OREB%) (https://www.cbaleague.com/) [20]. Each basketball player is represented as a node, and the connections (edges) between nodes indicate the similarity between players. The weight of the edges is calculated based on the performance variables between players, making this a weighted network. Due to the high edge density in networks constructed based on similarity measures, which approaches a fully connected graph, a sparsification procedure is often employed to remove weaker connections and reduce the graph’s density, making it theoretically and computationally more manageable [21]. This process is accomplished by progressively eliminating the weakest links(Table 1).

**Table 1:** 2023 CBA playoff teams and their corresponding network names.

Team	Network Name	Team	Network Name
Beijing Shougang	N1	Shanghai Juss	N7
Guangdong Dongguan	N2	Shanxi Fenjiu	N8
Guangzhou Long-Lions	N3	Shenzhen Marco	N9
Jilin Northeast Tigers	N4	Jiangsu Kendia	N10
Liaoning Bengang	N5	Zhejiang Guangsha	N11
Shandong High-Speed	N6	Zhejiang Jinqiu	N12

Complex network theory encompasses numerous metrics that characterize network features, with node degree and strength being the most fundamental and typical metrics to intuitively reveal the importance of various regions within the network [22]. Other metrics, such as betweenness centrality, serve as a global geometric measure, representing whether the shortest paths pass through a node, reflecting the role and influence of nodes or edges in the network, and emphasizing the node’s regulatory ability and mediating effects, though practical applications often focus more on a node’s control power [23]. Furthermore, network density, which describes the intensity of interconnections between nodes, is commonly used to measure the density and evolutionary trends of social networks [24]. Thus, this study analyzes the network’s small-world and scale-free properties based on topological metrics such as degree, betweenness, network diameter, average path length, and clustering coefficient [25–29]. We explore the network’s resilience from two perspectives: network efficiency and connectivity, and in two contexts: random attacks and deliberate attacks [30].

(1) The method for calculating similarity is:


$$w_{ij}=\text{1}-\frac{d_{ij}-min(d_{ij})}{max(d_{ij})-min(d_{ij})}$$ (1)

Here, $d_{ij}$ is defined as the Euclidean distance between player i and player j, normalized by the “four factors.” Initially, the dataset included 152 players. Players who averaged less than five minutes of playing time per game were excluded, leaving a final count of 135 players.

(2) Node degree:The degree of a node $k_{i}$ refers to the number of edges directly connected to node i. Generally, the degree of a node is positively correlated with its influence within the network [31]. Node degree, defined as the set of edges connected to a node, is the total number of regions that have trade relations with the region corresponding to that node. The formula for calculating the node degree, which is the number of adjacent edges to a node, is as follows:


$$\text{k}=\frac{\text{1}}{\text{n}}\sum_{\text{i}=\text{1}}^{\text{N}}{\text{K}}_{\text{i}}$$ (2)

N represents the number of nodes in the network, and the formula indicates the average node degree. Intuitively, the greater the degree of a node, the more important its position in the network.

(3) Node betweenness refers to the proportion of the shortest paths within the network that pass through a given node [32]. In the network, a higher node betweenness signifies a greater importance of that node in the network. Node betweenness is represented as:


$$B_{i}=\sum_{j,k\in V}\frac{N_{jk}(i)}{N_{jk}}$$ (3)

${\text{N}}_{\text{jk}}$ represents the number of shortest paths between node ${\text{V}}_{\text{i}}$ and node ${\text{V}}_{\text{k}}$; ${\text{N}}_{\text{jk}}(\text{i})$ denotes the number of shortest paths between node ${\text{V}}_{\text{i}}$ and node ${\text{V}}_{\text{k}}$ that pass through node ${\text{V}}_{\text{i}}$. Therefore, the greater the betweenness of a node, the more significant its position in the network.

(4) The clustering coefficient of a node is defined as the ratio of the actual number of edges between its adjacent nodes to the maximum possible number of edges among them. The distribution of clustering coefficients reflects the intensity of connections among players in the network [33], and is represented as:


$$C_{i}=\frac{\text{2}N_{i}}{k_{i}\left(k_{i}-\text{1}\right)}$$ (4)

$N_{i}$ is the actual number of edges among the nodes adjacent to node i; $k_{i}$ represents the degree of node i, which is the number of nodes adjacent to node i.

(5) The average path length L:represents the average value of the distances between any two nodes [34]. It reflects the degree of separation among players. Generally, the shorter the average path length, the tighter the connections between the players.


$$\text{L}=\frac{\text{2}}{\text{N}(\text{N}-\text{1})}\sum {\mathrm{\backslash nolimits}}_{\text{i}\neq \text{j}}{\text{d}}_{\text{ij}}$$ (5)

N represents the total number of nodes in the network; ${\text{d}}_{\text{ij}}$ denotes the shortest path length between nodes i and j.

(6) Resilience Analysis Metrics: Network Efficiency. Network efficiency is generally defined by the distance of the shortest paths. The lower the network efficiency, the higher the network’s vulnerability [35]. Network efficiency is defined as the average of the reciprocals of the shortest path lengths between two players, essentially summing up the risk efficiency of all nodes in the network:


$$\text{E}\equiv \left\langle \frac{\text{1}}{{\text{d}}_{\text{ij}}}\right\rangle =\frac{\text{1}}{\text{N}(\text{N}-\text{1})}\sum {\mathrm{\backslash nolimits}}_{\text{i},\text{j}\in \text{V},\text{i}\neq \text{j}}\frac{\text{1}}{{\text{d}}_{\text{ij}}}$$ (6)

Thus, the network efficiency effectively reflects the ease of energy flow within the basketball network. Higher network efficiency indicates better connectivity and, consequently, stronger resilience of the network.

(7) Network Connectivity:When nodes fail or are damaged, the network may be divided into subnetworks that cannot communicate with each other. The largest subnetwork, in terms of node count, is known as the largest connected component. The size of the largest connected component refers to the number of nodes in this subgraph. Network connectivity is defined as the ratio of the size of the largest connected component to the total number of nodes in the original network [36]. The formula is as follows:


$$\text{C}=\frac{\text{n}}{\text{N}}$$ (7)

C represents the maximum connectivity of the network, where n is the number of nodes in the largest connected subgraph of the basketball team network, and N is the total number of nodes in the network. The network is fully connected when C = 1; when C ≈ 0, the network is nearly paralyzed, with all nodes being isolated. It is evident that the larger C is, the better the network’s resilience.

## Results

### Analysis of network topological characteristics

#### Analysis of team complex network structures.

**Fig 1:**
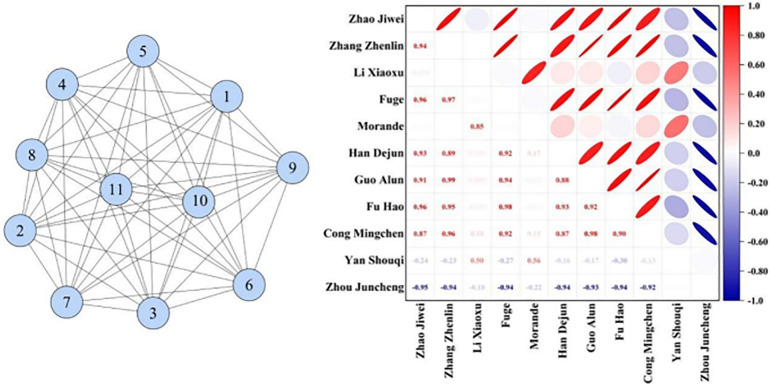
Network Topology of Liaoning Bengang.

**Fig 2:**
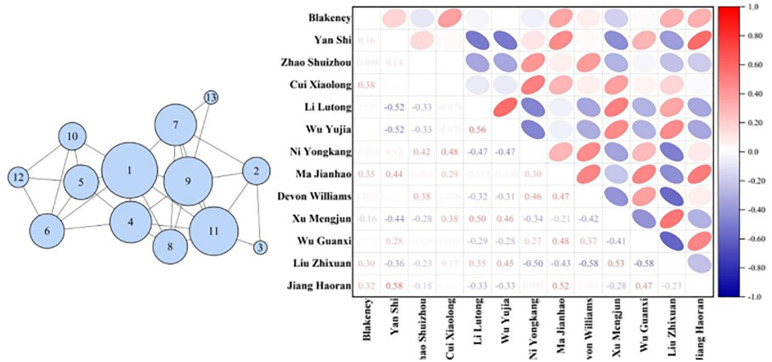
Network Topology of Jiangsu Kendia.

By analyzing the similarity networks of various teams, it is evident that team network structures can be classified into two categories: those with uniformly distributed node degrees, exemplified by Liaoning Bengang, Zhejiang Chouzhou Bank, and Zhejiang Dongyang Guang, and those with uneven node degree distributions, represented by the remaining nine teams. These characteristics provide a deeper understanding of the tactical styles of the teams. The subsequent analysis utilizes Liaoning Bengang (Fig 1) and Jiangsu Kendia (Fig 2) as case studies. The node degree within the networks of these two teams reveals significant differences. Node degree refers to the number of other nodes (in this context, other players) to which a node player is directly connected (Fig 1).

All players on the Liaoning Bengang team have a node degree of 11, indicating that each player has equal interaction opportunities with all other players during the game. This uniform pattern of interaction suggests that the team’s coordination is very balanced, with each player having a similar role and level of participation on the court. From a tactical perspective, this arrangement increases the team’s unpredictability, making it difficult for opponents to devise specific defensive strategies against one or a few players.In contrast, the node degree distribution among Jiangsu Kendia players varies from 4 to 10. This indicates that within the team, some players undertake more passing and organizing duties during the game, while others are less involved in these activities. This variation highlights a clear tactical arrangement, such as the differentiation between key players and support players, and further suggests that certain players have a more significant technical or tactical status within the team.

#### Node degree and degree distribution.

Node degree reflects the frequency of interaction among players within a team; a higher degree indicates frequent interactions and tighter team collaboration. Some teams show a high degree of consistency in node degree (for example, all players on Liaoning Bengang have relatively high degrees), which indicates a uniform level of cooperation within the team, with each player actively participating in the execution of team strategies. In contrast, some teams exhibit a wide range of node degrees (such as Jiangsu Kendia, with some players having a node degree of 4 and others as high as 10). This significant variation suggests a clear distinction between tactical cores and supporting roles within the team, or it may indicate that some players play a far more critical role on the field than others.

For teams with uniformly distributed node degrees, this indicates that the team employs a collective strategy on the court, not overly reliant on any single player. This style is beneficial for maintaining overall team stability and unpredictability. Teams with a wide range of node degrees, such as those where some players have as low as 2 or 3 while others reach 10 or more, likely have one or two core players, with others supporting these key figures. Such reliance on single or a few core players can be highly effective when the core players are in good form but also makes the team vulnerable to fluctuations in the performance of these key players.

**Fig 3:**
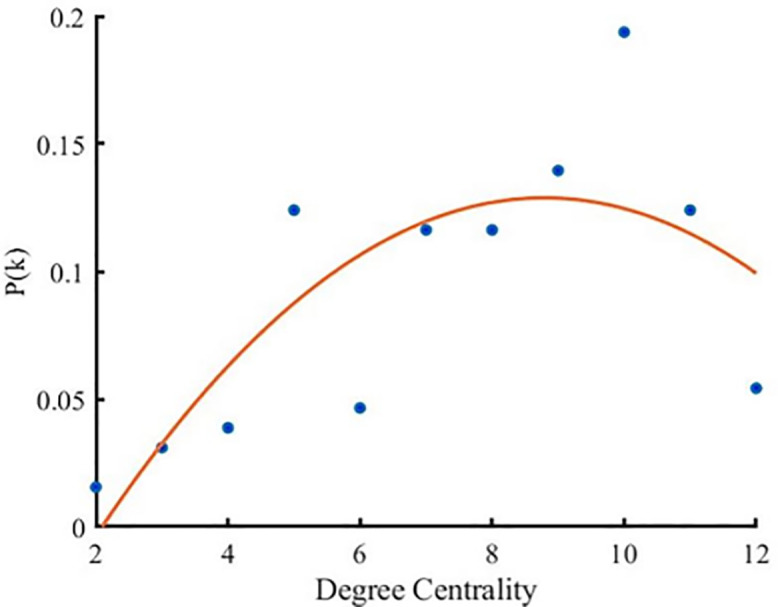
Node Degree Distribution for Twelve Teams.

For teams with evenly distributed node degrees, coaches should continue to maintain equal interaction among players, ensuring that every player can take on responsibilities when necessary. For teams with clear core players, coaches need to consider how to reduce dependence on individual players to enhance overall tactical flexibility and the team’s ability to respond to emergencies (Fig 3).

#### Node betweenness.

**Fig 4:**
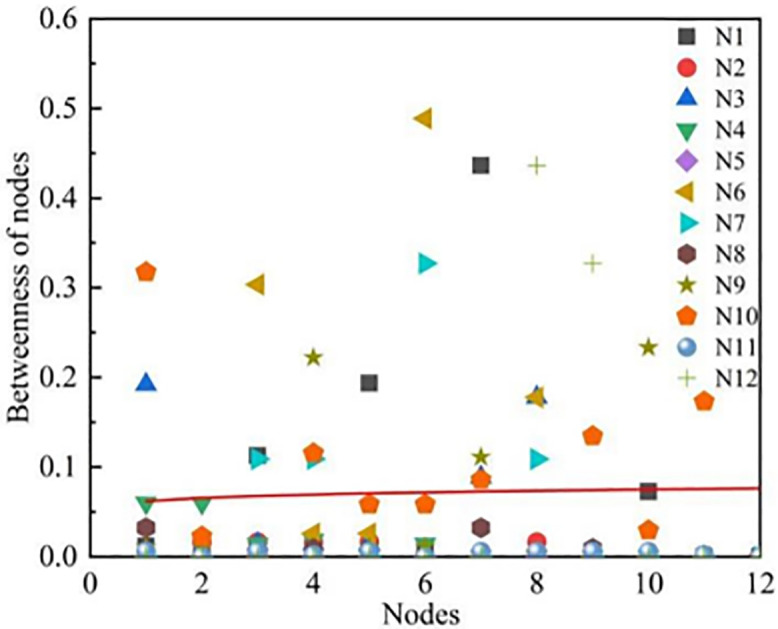
Node Betweenness Distribution for Twelve Teams.

In the constructed basketball networks, node betweenness primarily represents a player’s intermediary role or criticality within the team’s passing network. Specifically, it reflects the importance of a player on the paths of passes among all players. The higher a player’s node betweenness, the greater their role as a bridge in connecting other players or facilitating the flow of the ball, which typically indicates that the player plays a central role in the team’s tactics. It was found that teams such as Guangdong Dongguan Dayi, Jiu Tai Agricultural Bank, Liaoning Bengang, Shanxi Fenjiu, and Zhejiang Chouzhou Bank have node betweenness ranging from 0 to 0.1, with Liaoning Bengang having a node betweenness of 0 for every node. This suggests that these networks are close to a fully connected network, where each node is directly connected to every other node. Consequently, the shortest path length between any two nodes is 1, without the need to pass through a third node. In the networks formed by teams like Beijing Shougang, Shandong High-Speed, Shanghai Juss, and Zhejiang Dongyang Guang, there are nodes with node betweenness higher than 0.3. The higher the node betweenness of a player, the greater their role in connecting other players or facilitating the flow of the ball, which typically indicates that the player plays a central role in the team’s tactics (Fig 4).

#### Clustering coefficient and average path length.

Based on the statistical data of the clustering coefficients and average path lengths for twelve teams, it is possible to analyze the internal network structure and efficiency of these teams. The clustering coefficient reflects the closeness of nodes within a network; specifically in basketball, it indicates the tightness of passing between players and the presence of small groups. A higher clustering coefficient suggests more intense interactions and collaboration among players within a team. Teams with high clustering coefficients (for example, Zhejiang Dong yang Guang with 0.8974, Shanxi Fen jiu with 0.9067) exhibit high levels of cooperation and frequent interactions among players, indicating that the team employs a collective strategy, relying on tight formations and multiple passes for offense and defense. Teams with low clustering coefficients (for example, Guangzhou Long-Lions with 0.5986, Shenzhen Marco Polo with 0.6727) have relatively fewer interactions among players, with tactics more focused on individual players and direct attacks. The average path length indicates the average “distance” between any two nodes (players) in the network. A smaller value suggests more direct connections between players, facilitating faster movement of information or the ball. Teams with short average path lengths (for example, Shanxi Fen jiu with 0.6936, Liaoning Ben gang with 0.7219) have direct and fast passing routes between players, which are characteristic of efficient, fast offenses and compact defenses. Teams with longer average path lengths (for example, Jiangsu Kendia with 1.1681, Shandong High-Speed with 1.1513) indicate that more intermediaries are needed in passing and collaboration, which slows down the attack speed but also increases the difficulty of prediction for opponents (Table 2).

**Table 2:** Clustering coefficients and average path lengths for each team.

Team	Clustering Coefficients	Average Path Length
Beijing Shou gang	0.8484	1.1231
Guangdong Dongguan	0.8333	0.7586
Guangzhou Long-Lions	0.5986	0.9207
Jilin Northeast Tigers	0.715	0.7637
Liaoning Bengang	0.8308	0.7219
Shandong High-Speed	0.8212	1.1513
Shanghai Juss	0.8385	0.9919
Shanxi Fenjiu	0.9067	0.6936
Shenzhen Marco	0.6727	0.9614
Jiangsu Kendia	0.7418	1.1681
Zhejiang Guang sha	0.806	0.7228
Zhejiang Jinqiu	0.8974	1.0915

#### Analysis of network resilience metrics.

According to complex network theory, simulating random and deliberate attacks on the complex networks of CBA teams is of significant research importance and practical value. These simulations help researchers and managers understand the network’s robustness and vulnerability in the face of potential threats and disruptions, as well as its structural characteristics.

#### Random attacks.

**Fig 5:**
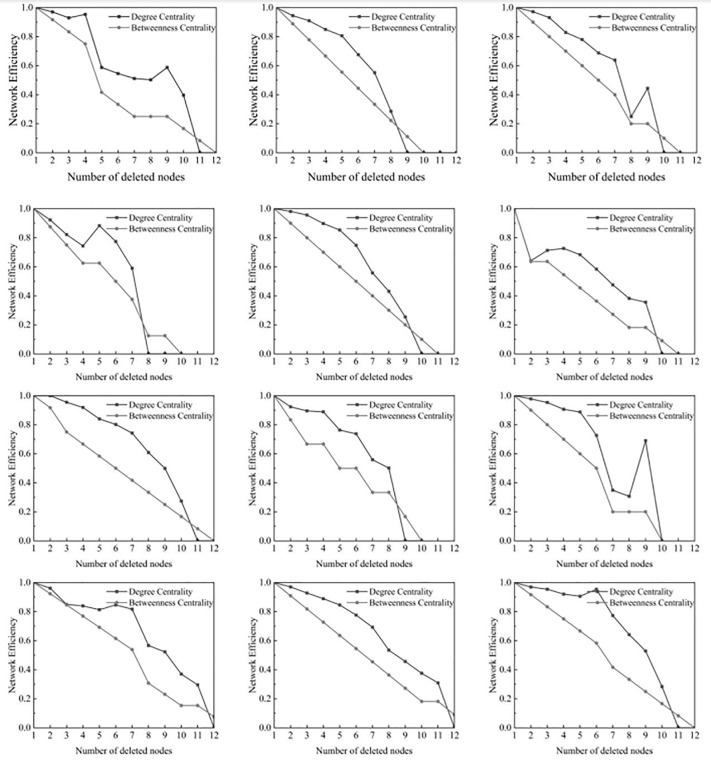
Network Efficiency and largest connected component under random attack and targeted attack based on degree or betweenness centrality.

Nodes within the network are removed at random. This type of attack simulates node failure due to random events, such as a player’s accidental injury or unexpected departure from the team. As shown in (Fig 5), during the early stages of a random attack, most teams’ network efficiency remains relatively high, close to a value of 1, indicating that the network is highly connected at the outset, allowing information or strategies to be quickly disseminated among players. As nodes continue to be removed, the network efficiency of most teams begins to gradually decline. This reflects the impact of losing key nodes (core players or players in critical positions) on the overall network structure. Some teams (such as N8, N9) experience a sharp decline in network efficiency after a certain number of nodes are removed, even dropping to 0. This indicates that these teams are extremely reliant on a few key players, and the absence of these players due to injury or other reasons greatly hinders the overall tactical operation of the team. In rare cases (such as N1, N9), network efficiency rebounds after an initial decline, due to the formation of new connectivity paths among the remaining nodes, suggesting that teams can partially restore efficiency by adjusting tactics or enhancing the roles of other players in the absence of key players.

Initially, the maximum connectivity of all 12 teams is 1, meaning the entire network is fully connected at the start (Fig 5). As nodes are randomly removed, the maximum connectivity of nearly all teams gradually decreases. This indicates that network connectivity is weakening, reflecting the increased vulnerability of the team’s structure as random nodes are lost. Some teams experience a faster decline in connectivity (such as N5, quickly dropping from 1 to 0.3636), while others decline more slowly (such as N11, gradually falling to 0.0909). This reflects differences in the internal structure of the teams; teams that decline quickly rely on a few key nodes, while those that decline more slowly have a more balanced structure or better redundancy. Some teams experience fluctuations in connectivity during the decline (N9, where connectivity initially decreases and then slightly increases), due to the presence of multiple large connected components within the network, which change in relative size as nodes are removed. Ultimately, the connectivity of all teams will drop to 0, indicating that the network has become completely disconnected.

#### Deliberate attacks.

Deliberate attacks target key nodes in the network, such as those with the highest betweenness or degree. In basketball networks, this involves the strategic absence of key players or tactical cores, such as intentionally reducing the playing time of a main player for tactical reasons.

Attacks based on node degree assume that highly connected players (i.e., those central or well-connected within the team) are removed first. This analysis is crucial for understanding the importance of key players and their contributions to the stability of the team structure. Nearly all teams start with very high network efficiencies, close to or equal to 1, indicating that all players are highly connected within the network initially, allowing for rapid dissemination of information and tactics. During the attack, teams can be categorized by the rate of network efficiency decline.

Some teams experience a rapid decline in network efficiency. These teams’ efficiencies drop sharply after the initial few rounds of attacks, indicating a high dependency on a few key players. When these critical nodes are removed, the entire team’s tactical operations and information flow are severely impacted. For example, Team N5’s efficiency plummets from 1 to 0.273, showing extreme reliance on key nodes. This steep decline indicates an overreliance on certain core players, and the team’s overall performance suffers significantly if these players are unable to participate. Team N8’s efficiency drops straight from 1 to 0.2027 and then quickly to zero, reflecting a rapid collapse of the team structure once key nodes are removed. A similar situation occurs with Team N10, where efficiency quickly drops to 0.1889, indicating similar structural vulnerabilities.

Other teams exhibit a gradual decline in network efficiency. These teams have a slower rate of efficiency loss, indicating a more balanced player structure or the ability to maintain good tactical execution even after key players are removed. For example, Team N1 shows a more gradual efficiency decline from 1 to 0, indicating better structural robustness and lower dependency on individual nodes. Team N2’s decline, although from 0.999 to 0, is overall more gradual, showing that the team maintains some structural integrity even after multiple attacks. Team N3’s efficiency slowly drops from 0.8998 to 0, with a relatively slow rate of decrease, suggesting good redundancy and diverse tactical arrangements. Team N12 demonstrates strong robustness, gradually declining from 1 to 0 without any sharp drops. Some teams, after experiencing an initial rapid decline, stabilize their efficiency (like Teams N1 and N6). This indicates that even though some important players are removed, other players in the team can take on more responsibility, or the team tactically adjusts well to the new player structure. Eventually, all teams’ efficiencies drop to zero, indicating complete loss of connectivity and the inability to execute tactics effectively. This typically occurs faster in smaller teams or those relying on a very few top players.

**Fig 6:**
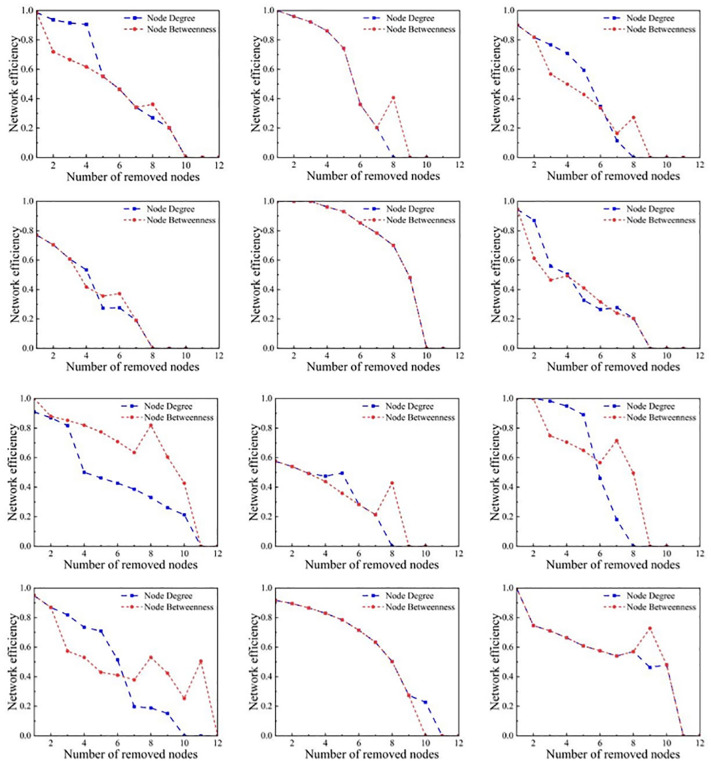
Changes in Network Efficiency Under Maximum Degree Disruption and Maximum Betweenness Disruption.

In attacks based on node betweenness, the most critical nodes are usually removed first. This strategy tests the team’s ability to cope with the loss of core strengths. Initially, all teams have very high network efficiencies, with most teams’ efficiencies close to or equal to 1, indicating that information and tactics are very smoothly transmitted within the team before the attacks. Teams that show a rapid decline (such as Teams N3, N4, N6): These teams see a swift drop in network efficiency after just a few rounds of deletions, displaying a high dependence on certain key players. Particularly, Team N6, after key nodes are deleted, sees efficiency quickly fall from 0.944 to 0.3159, showing that these key players are pivotal to the entire team’s tactical operations. Relatively slow declining teams (Teams N1, N2, N12): These teams also see a decline in efficiency but at a more gradual rate, indicating a more balanced player structure, not solely dependent on individual key players, or possessing good tactical adaptability and player depth. A few teams, such as N5 and N11, stabilize or even slightly recover in efficiency after an initial decline, due to strong synergies among remaining players or new tactical adjustments. Notably, Team N10 shows extreme data fluctuations, with efficiency suddenly spiking to 1 at one point during the simulation, due to a unique network restructuring phenomenon (Fig 6).

When analyzing the maximum connectivity data under node degree deletion, a deeper understanding of the robustness and connectivity of the network structures of various CBA teams after losing key nodes is gained. The maximum connectivity of the teams under node degree attacks shows varying trends: all teams start with a maximum connectivity of 1, indicating that all players within each team are fully connected before any nodes are removed. As key nodes are progressively removed, the maximum connectivity of each team begins to gradually decrease. Teams with rapid declines (such as N6, N12) exhibit a swift decrease in maximum connectivity, indicating a team structure that relies on a few key players. Once these players are missing, the entire team’s structure and tactical execution capabilities quickly collapse. Relatively stable teams (such as N2, N11) show a more gradual decline, suggesting a more balanced player structure.

**Fig 7:**
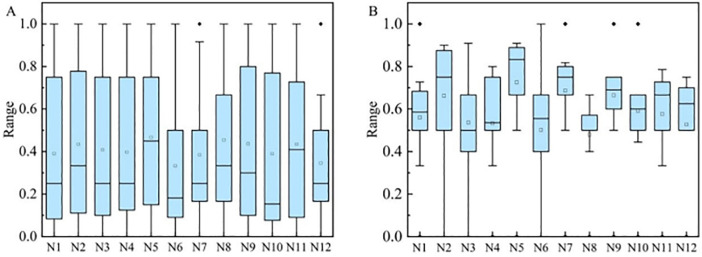
Changes in Maximum Network Connectivity Under Maximum Degree. Disruption and Maximum Betweenness Disruption.

All teams initially have a maximum connectivity of 1, indicating that all members of the team are in a fully connected state at the start. As nodes with high betweenness are gradually removed, the maximum connectivity of most teams gradually decreases. This decline reflects the importance of key nodes in the overall structural connectivity of the team network. Teams with rapid declines (N7 and N10) show a quick reduction in maximum connectivity after just a few rounds of deletions, highlighting a high dependence on certain core nodes to maintain network connectivity. N10’s maximum connectivity quickly drops from 1 to 0.5, indicating the network splits into smaller subnetworks after critical nodes are removed. Teams with slow declines (N1, N2, N5) have a relatively gradual decrease in maximum connectivity. This slow decline suggests a more dispersed team structure. Relatively stable teams, such as N8 and N9, maintain a relatively high level of maximum connectivity for a long duration during the deletion process, indicating better structural redundancy and stronger network connectivity (Fig 7).

## Discussion

This study applies complex network theory to conduct a detailed analysis of the network structures of 12 teams in the 2023 CBA playoffs. The results demonstrate that a team’s network connectivity and resilience are closely linked to the balance of its structure. For instance, Liaoning Ben gang’s team, with a uniform node degree distribution (node degree of 11), a high clustering coefficient (0.83), and a short average path length (0.72), exhibits strong network connectivity and resilience. This structural advantage enables the team to maintain high operational flexibility and tactical diversity even when key players are limited. Conversely, teams like Jiangsu Kendia, with uneven node degree distributions, show significant structural vulnerability when key nodes are compromised.

Compared to previous studies, such as those by Manuel which primarily focused on analyzing the centrality of individual players in a team without exploring how such centrality impacts the entire network’s resilience [37], this study, by incorporating metrics like node degree and clustering coefficient, reveals more complex network dynamics. Furthermore, unlike Fewell’s research, which used passing data to analyze network patterns in basketball games focusing on the passing structure of a single game [38], this study starts from a dataset of the playoffs as a whole, revealing the long-term network structural stability and resilience of the teams. Although studies like those by Kempe have used Self-Organizing Maps and Dynamic Controlled Neural Networks to identify tactical patterns, these methods have limitations in directly measuring a team’s network resilience [39]. This study, by directly analyzing structural features of networks, can more accurately predict a team’s responsiveness to sudden events such as key player injuries. Vaz et al. through social network analysis of soccer teams’ collaboration networks, emphasized the correlation between network centrality and team success [40]. This study expands on this perspective, not only focusing on network centrality but also examining other topological features like clustering coefficients and path lengths, thus providing a more comprehensive framework for analyzing team performance and resilience.

The main innovation of this study lies in the extensive application of complex network analysis methods to the study of topology and resilience in CBA teams, particularly in quantifying team efficacy and structural robustness. By utilizing multiple network metrics such as node degree, clustering coefficient, and average path length, this study reveals the intrinsic link between team structure and its resilience, highlighting the importance of optimizing network structural balance. Theoretically, the characteristics of small-world networks, such as local tight cooperation and global tactical connectivity, and the roles of key players in scale-free networks, provide a pathway to deeply understand how these structures affect team performance.

While this study has yielded noteworthy results, it also presents limitations, particularly regarding the scope and depth of the research data. The dataset is confined to 12 teams from the 2023 CBA playoffs, which may limit the generalizability and applicability of the findings; further replication in the NBA or other leagues is necessary to assess universality. Additionally, the study is based on existing game statistics and does not account for dynamic in-game factors, such as players’ psychological states and real-time tactical adjustments. Edges were constructed based on performance similarity rather than on-court spatial roles, leaving positional tactics unexplored. Furthermore, the similarity network was sparsified by iteratively removing the weakest 5% of edges until the density reached 0.15—an approach that, while stable, has not been compared with more advanced backbone algorithms. Lastly, the cross-sectional design compares different teams but lacks longitudinal or case-control contrasts within teams (e.g., evaluating the same squad with and without a key player), thus the causal impact of individual athletes on network resilience remains inferential. Future research should incorporate multi-season tracking data, permutation-based null models, and position-informed layers to quantify each player’s unique contribution to team topology and, ultimately, to win-loss records.

## Conclusion

Through complex network analysis of the internal network structures and resilience of CBA teams, this study not only provides robust scientific support for basketball tactical planning and team management but also opens new perspectives and methods for the study of other team sports. These findings help to advance the scientific development and tactical innovation of basketball and other team sports.

## Supporting information

S1 Dataset Raw data of player performance indicators for the 2023 CBA playoff teams. (RAR)
